# Effect of Red Light-Emitting Diodes Irradiation on Hemoglobin for Potential Hypertension Treatment Based on Confocal Micro-Raman Spectroscopy

**DOI:** 10.1155/2017/5067867

**Published:** 2017-01-12

**Authors:** Xuejun Qiu, Hanchuan Huang, Zhitong Huang, Zhengfei Zhuang, Zhouyi Guo, Songhao Liu

**Affiliations:** ^1^MOE Key Laboratory of Laser Life Science & SATCM Third Grade Laboratory of Chinese Medicine and Photonics Technology, College of Biophotonics, South China Normal University, Guangzhou, China; ^2^Guzhen Productivity Promotion Center, Zhongshan, China

## Abstract

Red light-emitting diodes (LED) were used to irradiate the isolated hypertension hemoglobin (Hb) and Raman spectra difference was recorded using confocal micro-Raman spectroscopy. Differences were observed between the controlled and irradiated Hb by comparing the spectra records. The Raman spectrum at the 1399 cm^−1^ band decreased following prolonged LED irradiation. The intensity of the 1639 cm^−1^ band decreased dramatically in the first five minutes and then gradually increased in a time-dependent manner. This observation indicated that LED irradiation increased the ability of oxygen binding in Hb. The appearance of the heme aggregation band at 1399 cm^−1^, in addition to the oxygen marker band at 1639 cm^−1^, indicated that, in our study, 30 min of irradiation with 15.0 mW was suitable for inhibiting heme aggregation and enhancing the oxygen-carrying capacity of Hb. Principal component analysis showed a one-to-one relationship between irradiated Hb at different time points and the corresponding Raman spectra. Our approach could be used to analyze the hemoglobin from patients with confocal micro-Raman spectroscopy and is helpful for developing new nondrug hypertension therapy.

## 1. Introduction

Hypertension is a global public-health challenge [1]. According to the report of the World Health Organization (WHO) in 2012 [2], one-third of the adult population is affected by this noncommunicable disease. Since hypertension is highly prevalent, it presents a major risk factor for many common underlying causes of diseases, including cardiovascular (CVD) [3, 4], stroke [5, 6], and kidney disease [7, 8]. Clinical manifestation of hypertension includes elevated arterial blood pressure and a weakened capacity for oxygen transport by erythrocytes, which would lead to the above listed diseases.

Common methods of treating hypertension include drug intervention [9, 10] and nondrug therapy [11, 12]. Benefits of drug intervention on hypertension have been well documented, especially in high-risk individuals. However, treated patients that are affected by hypertension still have higher rates of hypertension-related CVD complications. This anomaly might result from the failure to radically cure hypertension, residual target organ damage such as left ventricular hypertrophy (LVH), or both [4]. Thus, drug intervention is not an optimal choice for hypertension treatment, and the disease pathogenesis of hypertension remains largely unknown [13]. Understanding the underlying molecular mechanisms of hypertension has aroused increasingly interests from clinicians and scientists.

Erythrocyte plays a crucial role in the human body for their prolific ability to transport oxygen and carbon dioxide to different tissues. The most important component in erythrocytes is Hb, which is the primary oxygen-transport protein in humans. However, limited efforts have been applied to determine whether the Hb structure could be changed at the molecular level in erythrocytes of the hypertensive patient. Previous studies have found a positive association between Hb levels and the relative risk of hypertension [14]. Anand et al. [15] and Lipšic et al. [16] used erythropoietin treatment to recover the levels of Hb, which appeared to improve patient outcome in the settings of chronic heart failure or kidney disease. Thus, identifying changes in the molecular structure of Hb in hypertension and recovering it could be potentially helpful for treating hypertension-related disease. However, as mentioned above, drug intervention in hypertension-associated diseases is limited. Therefore, we have attempted to seek a novel nondrug therapy to recover the normal functional ability of Hb in transporting molecular oxygen.

There are several reports documenting significant positive effects of low-intensity laser therapy (LILT) on biological systems [17–20]. In recent* in vitro *studies on cell culture models, data was provided demonstrating the effects of low-level lasers in muscle, bone, skin, and certain tumors and in sperm and the biostimulatory effects on organisms at the molecular level [21]. The most commonly used laser in many LILT studies is the Helium-Neon (He-Ne, *λ* = 632.8 nm) laser. In this study, we utilized red LED as a replacement of reduced low-intensity laser to irradiate erythrocytes in hypertension patients* in vitro*. LED typically consume low levels of power and are essentially heat-free. As a relatively mature technology, LED have been widely used in clinical treatment [22–25], and results have shown promising effects in these diseases. To our best knowledge, there are only few reports describing LED therapy on hypertension. In this study, after irradiating hypertension erythrocytes* in vitro*, we expected an inhibition of heme aggregation, a reduction of the metHb content, and an increase of the oxygen-carrying capacity of Hb. In order to analyze the molecular changes of Hb after irradiation, a robust tool was urgently needed to characterize the levels of Hb and to provide new information of hypertension treatment at the molecular level.

Confocal micro-Raman spectroscopy is a potent tool in biomedical diagnostics due to its high sensitivity, increased capacity to be multiplexed, and its robustness. Confocal micro-Raman spectroscopy is rapid, on-site, and specific and only requires a minimal sample size, which can be used as a detection tool in blood and other biological matrices [26]. In comparison with other diagnostic technologies [27, 28], it can measure molecular vibrations and provide fingerprint signatures of various tissue biomolecules. As this technique is free from water interference, it can also be applied to diagnostic studies* in vivo*. In this study, we utilized confocal micro-Raman spectroscopy to identify molecular changes in irradiated Hb and to establish the relationship between micro-Raman spectra and hypertension LED-mediated therapy.

## 2. Materials and Methods

### 2.1. Sample Preparation

Following standard protocols, fresh peripheral venous blood (5 mL) was obtained by venipuncture from a patient with hypertension CVD and placed in glass tubes that contained acid citrate dextrose as an anticoagulant. Blood samples were stored in an 8°C refrigerator immediately after collection until they were required for irradiation or clinical measurements. The blood was centrifuged (3000 rpm, 5 min, 4°C) to produce a buffy coat. The erythrocytes were then harvested from the base of the tube and washed three times with isotonic phosphate buffered saline (PBS) at pH 7.4.

**Figure 1:**
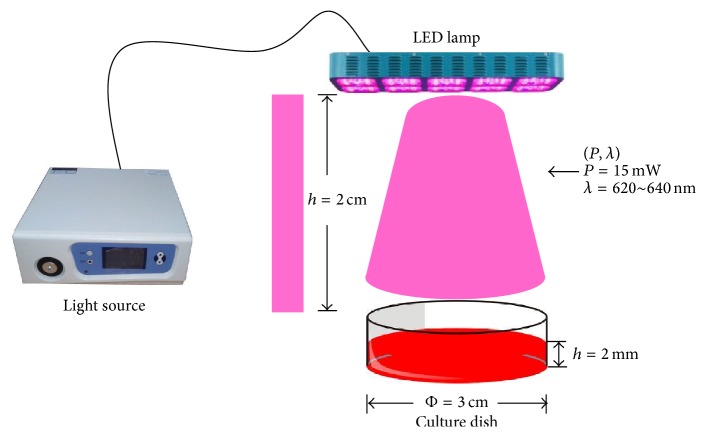
Schematic drawing of red light-emitting diode irradiation.

The erythrocytes were placed in a culture dish with a diameter of 3 cm, wherein the depth of the erythrocyte specimens was about 2 mm. The distance between the LED lamp and the specimens was adjusted to 2 cm, and the LED were applied vertically with a continuous power output of 15.0 mW (irradiance: 2.1 mW/cm^2^) based on a previously published study [17]. The central wavelength of red LED was 620–640 nm, which was comparable to the He-Ne laser device with a wavelength of 632.8 nm [21]. Different irradiation times were obtained by setting the timer to automatically turn off the power. The samples were irradiated for 5, 10, and 30 min, respectively. The schematic drawing of red LED irradiation is shown in Figure 1. This irradiation device is made up of the control unit and array LED. The control unit could adjust the irradiation power and time. And the array LED is made up of 10 red LED beads (ShenZhen GeTian Optoelectronics Co., LTD, China) which are of super high flux output, high luminance, and low thermal resistance. The detailed specification and parameters of LED are depicted in Table 1. Afterwards, the isolated Hb was extracted from the irradiated erythrocytes.

In order to eliminate the impact of the erythrocyte membrane and investigate the effect of LED irradiation on Hb directly, the isolated Hb specimens were prepared by adding the irradiated erythrocytes to double distilled water at a volumetric ratio of 1 : 1. Next, the hemolytic erythrocytes were centrifuged at 3000 rpm for 30 min at 4°C. The supernatants were carefully separated and used as Hb samples, which were then placed in glass capillary tubes with a diameter of approximately 1 mm for Raman detection. We obtained the full local approval from the ethics committee of the university for all of the experiments described above.

### 2.2. Raman Microspectrometer

A Renishaw inVia confocal micro-Raman microscope was equipped with an ×50 objective lens (NA = 0.75) and a laser excitation of 514 nm was used to obtain all spectra. The resolution of this instrument was 1 cm^−1^ and the laser power that was focused on the samples was 0.15 mW. Each spectrum was recorded with a 3 s exposure time and five accumulations. In our experiment, the 514 nm diode laser with a power of 0.15 mW had minimal effects of thermal degradation and photo degradation. The back-scattered Raman light with a recorded range of 600–1800 cm^−1^ was collected with a slit-width in our experiment of approximately 50 *μ*m. Prior to initiating Raman scanning, the silicon wafer with a 520 cm^−1^ band was used for frequency calibration. All of the data were collected under the same conditions.

### 2.3. Atomic Force Microscopy (AFM)

All erythrocyte samples were deposited on freshly cleaved mica surfaces and fixed with 2.5% glutaraldehyde for 5 min. The fixed cells were washed five times with distilled water in order to avoid the disturbance of salinity and then air-dried at room temperature. AFM (AutoProbe CP, Veeco Instruments, Santa Barbara, USA) test was carried out in air and imaged in contact mode. Microfabricated silicon cantilevers with a constant force of approximately 2.8 N/m were used. AFM images were planar leveled using Proscan Image Processing Software Version 2.1 (Thermo Microscopes) provided with the instrument in order to eliminate low frequency background noise in the scanning direction.

## 3. Data Analysis

In order to conduct statistical analysis, at least 45 Raman spectra were randomly obtained from each Hb sample. The final Raman spectrum was taken as the baseline that was corrected by the software program R 2.8.1 provided by Renishaw. Afterwards, the data was smoothed, normalized, and averaged by ORIGIN PRO 8.0 (Origin Lab Corporation, Northampton, MA, USA) [29, 30].

Principal component analysis (PCA), which is a multivariate analysis, is developed in the Matlab platform (The Mathworks, Inc., Natick, MA, USA) [31]. PCA is a technique of data dimensional reduction by orthogonally projecting data onto a lower dimensional linear space such that the variances of the projected data are maximized. It is an efficient approach for data classification and statistical data analysis. In our study, PCA was utilized to retain the most important information for Hb diagnosis and characterization. The analysis was oriented towards modeling a variance-covariance structure of a data matrix from which the eigen values that corresponded to principal components were extracted. Each principal component (PC) was a linear combination of the *n* independent wave number variables *x*_1_, *x*_2_, *x*_3_, …, *x*_*n*_. For example,

(1) $$\begin{array}{c} \text{PC}1=\alpha_{1}x_{1}+\alpha_{2}x_{2}+\cdots +\alpha_{n}x_{n}. \end{array}$$

The first PC accounts for the greatest variance and thus corresponds to the largest eigen value. The second PC is orthogonal to the first PC, with each successive PC being orthogonal to all those preceding it, and this accounts for a decreasing proportion of the variance. In this paper, we chose to analyze the first three PC items.

## 4. Results

### 4.1. Raman Spectra of Hb from Healthy Person

**Figure 2:**
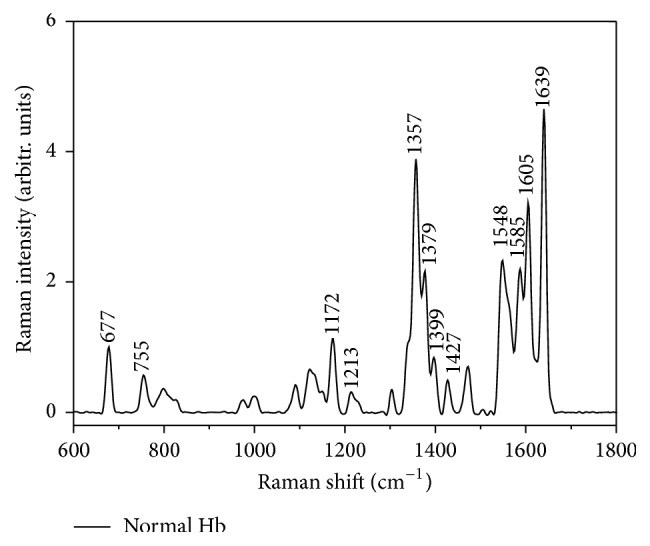
Averaged Raman spectra of normal hemoglobin from healthy person.

The averaged Raman spectra of Hb from healthy person are shown in Figure 2. The *ν*_7_ band at 677 cm^−1^ that is assigned to the (pyrrole) sym is used to normalize all Raman spectra because its intensity remains the same as that for Hb of healthy person. The assignments of all major Raman peaks and local coordinates for Hb from healthy person are depicted in Table 2.

The 810 cm^−1^ peak corresponds to the C_*m*_H out-of-plane deformation stretching mode, while the 1213 and 1226 cm^−1^ peak corresponds to the C_*m*_H in-plane deformation bending mode. The bands that are located between 1100 and 1400 cm^−1^ attribute mainly to the pyrrole in-phase half-ring breathing vibrations. The band at 1226 cm^−1^ is the marker of oxygenated erythrocytes while the band at 1213 cm^−1^ is indicative of deoxygenated erythrocytes [32]. Also they are well-known markers for oxy-deoxy state of Hb. It is assigned to *ν*_5_ + *ν*_8_ and *ν*_13_, respectively. The 1357 cm^−1^ peak is assigned to *ν*_4_. According to previously published studies [33, 34], the Raman peak at 1399 cm^−1^ (*ν*_20_) is the marker of heme aggregation. The 1585 cm^−1^ (*ν*_37_) peak corresponds to the C_*α*_C_*m*_ asymmetric stretching mode. The core-size or spin state marker band region lies between 1500 and 1650 cm^−1^. In particular, the appearance of the 1639 cm^−1^ (*ν*_10_) band, which has the local coordinate character of *ν*(C_*α*_C_*m*_)_asym_, is the characteristic of the O_2_ concentration marker in Hb.

### 4.2. Raman Spectra of Controlled and Irradiated Hb from Hypertension Person

**Figure 3:**
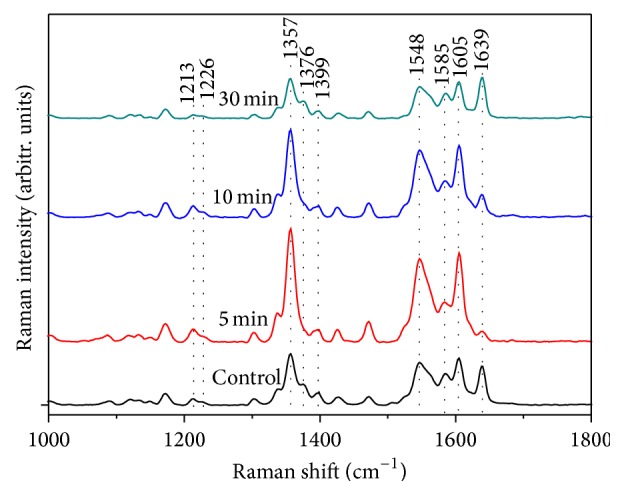
Raman spectra of controlled hemoglobin and irradiated hemoglobin from hypertension person.

**Figure 4:**
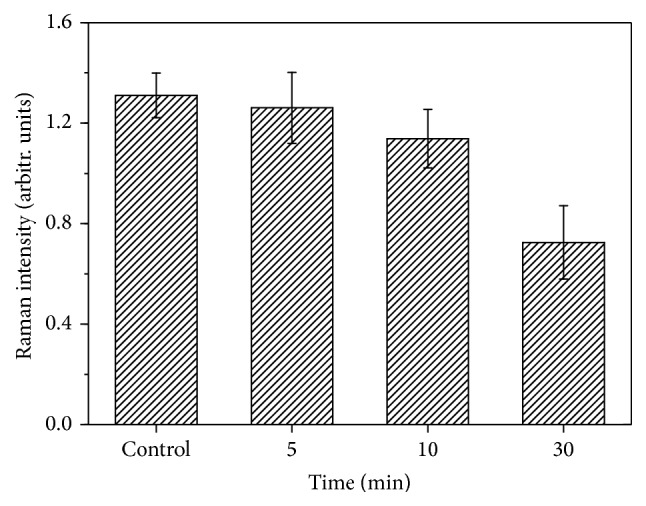
Quantitative analysis of Raman band (1399 cm^−1^) on irradiated hemoglobin from hypertension person.

Figure 3 shows the spectra of controlled Hb (CH) and irradiated Hb (IH) in hypertension samples. Both the CH and IH Raman spectra indicated an extreme ordering of the hemes within cells. Figure 3 reveals that the band at 1213, 1226, and 1399 cm^−1^ decreased with prolonged irradiation time. The quantitative analysis of 1399 cm^−1^ peak is shown in Figure 4. The intensities of the 1213, 1226, and 1399 cm^−1^ peaks decreased slightly in the first 10 min; then an obvious decline was observed after 30 min of irradiation. Since the 1226 and 1213 cm^−1^ bands are the markers for oxy-deoxy state of Hb, the decreased intensity of 1226 cm^−1^ and the comparable intensity of 1213 cm^−1^ compared to controlled Hb in our study perfectly presented the transition from oxygenated to deoxygenated state. Also the Raman peak at 1399 cm^−1^ is the marker of heme aggregation; the decreased intensity of 1399 cm^−1^ in our study indicated that heme aggregation was inhibited by prolonging the irradiation time. Obvious enhancements of intensity in the Raman bands at 1357, 1548, and 1605 cm^−1^ were observed after 5 min of irradiation. However, the intensity of bands at 1376, 1585, and 1639 cm^−1^ decreased conversely. According to previous reports, bands at 1357, 1548, and 1605 cm^−1^ were enhanced in deoxygenated Hb, while bands at 1376, 1585, and 1639 cm^−1^ increased in oxygenated Hb [35]. 1639 cm^−1^ is known as a symbolic band of O_2_ concentration. The decreased intensity of the 1639 cm^−1^band indicated that the oxygenated Hb trend was not obvious in the first 5 min of light therapy.

When the irradiation time was extended to 30 min, the intensities of the 1357, 1548, and 1605 cm^−1^ peaks were gradually decreased; however, the intensities of 1376, 1585, and 1639 cm^−1^ were gradually increased. The enhanced intensity seen at the 1639 cm^−1^ peak indicated that Hb could bind O_2_ more easily. Consequently, as with the marked peaks of oxygenated and deoxygenated Hb noted before [35], the oxygenated Hb content increased with prolongation of the irradiation time. However, after 10 min of irradiation, the intensity of the 1639 cm^−1^ band was still lower than that of the controlled band and exceeded the control level after 30 min of irradiation. The fluctuation of intensity at the 1639 cm^−1^ band indicated that the irradiated Hb had initially deoxygenated and then oxygenated during the overall irradiation process. This observation meant that a 30 min irradiation time with a power of 15.0 mW was suitable to inhibit heme aggregation and raising the oxygen-carrying ability of Hb. The extracted Hb molecules from the erythrocytes could introduce artifacts for the measurement of their Raman spectra.

### 4.3. Principal Component Analysis

**Figure 5:**
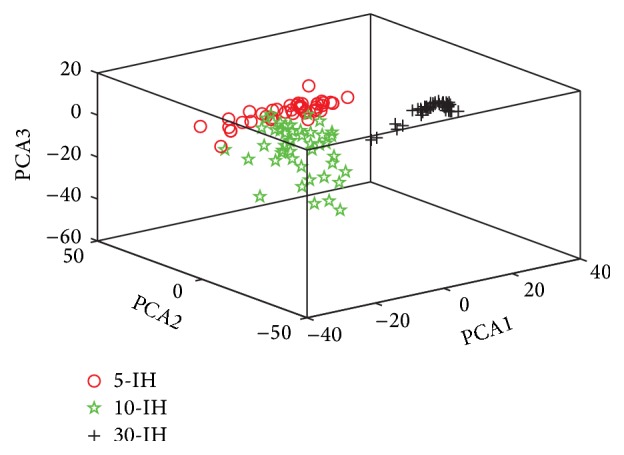
First three PC (PC1, PC2, and PC3) scores plots of 3D-PCA for irradiated hemoglobin from hypertension person (5-IH = irradiated Hb for 5 min, 10-IH = irradiated Hb for 10 min, and 30-IH = irradiated Hb for 30 min).

In this study, we chose the first three PC items to build a 3D-PCA [36]. The 3D plots of PC1, PC2, and PC3 that were calculated for Hb and irradiated for 5, 10, and 30 min, respectively, are presented in Figure 5. A total of 45 Raman spectra from each sample were used for 3D-PCA. The plots showed that almost all Raman spectra of 5-IH were located to the upper left of the box, and the 10-IH spectra were located to the middle. By contrast, the 30-IH spectra were located to the upper right of the box. This result shows that this was a good classification relative to other methods, and a one-to-one relationship was established for Hb in hypertension within different irradiation times and the corresponding Raman spectra. The excellent classification outcome seen in the 3D-PCA figure indicated that confocal micro-Raman spectroscopy provided a highly satisfactory approach to understand the molecular variations of irradiated Hb in hypertension. Confocal micro-Raman spectroscopy could be used to analyze the effects of hypertension treatment.

## 5. Discussion

Raman spectroscopic analysis is particularly attractive for biomedical purposes, especially given that it is an ideal tool in the detection and monitoring heme groups within single cells. In this study, we evaluated Raman spectral properties of IH with confocal Raman spectroscopy. The quantitative analysis depicted that the heme aggregation state was inhibited in IH spectra.

Hb catalyzes a variety of vital redox reactions in biological systems. It is largely dependent on the central iron- (Fe-) containing moiety, which could take on various types of oxidation and spin states [37]. The Hb consists of a porphyrin macrocycle with an extended *π* configuration. This porphyrin surrounds an Fe atom that is coordinated with four nitrogen atoms. In the deoxygenated state, the Fe atom is in the ferrous high spin state and the ferrous ion is combined with an H_2_O molecule below the porphyrin plane. While in the oxygenated state, the Fe atom is in a ferrous low spin state, and O_2_ will replace the H_2_O molecule. The changes in the electronic state are followed for Hb samples drawn at specific times during* in vitro* irradiation. Erythrocytes rely on the ferro-Hb to transport O_2_. However, metHb is in the ferric high spin state and thus cannot bind O_2_. In some cases, if the ferro-Hb are oxidized to metHb, they will lose the ability to transport oxygen. In this study, the oxygen marker band at 1639 cm^−1^ was increased obviously after 30 min LED irradiation. It indicated that the content of ferro-Hb was increased and oxygen-transporting ability of Hb was promoted after irradiation. The results of* in vitro* erythrocytes experiment confirmed that suitable LED irradiation conditions could alter the glycolysis of erythrocytes and the formation of ATP [38, 39], then preventing ferro-Hb from further oxidizing with metHb. Thus, LED irradiation could enhance the oxygen-carrying ability of erythrocytes and could be used to treat hypertension efficiently.

There are two main conformational states in Hb. Raman spectroscopy has already been shown to be a powerful technique for monitoring the molecular dynamics of the R-form (i.e., relaxed conformation with a higher O_2_ concentration) to the T-form (i.e., tense conformation with a lower O_2_ concentration) of Hb [40]. It was shown that the reversible photodissociation of Hb-ligand complexes, initiated by erythrocyte irradiation changed the oxyHb and deoxyHb levels, and the conformational transitions in the Hb macromolecules accompanying ligand detachment and addition were responsible for changes in their Raman spectra.

Figure 4 shows that the intensity of the 1399 cm^−1^ peak had decreased with the extension of the irradiation time, which is the heme aggregation marker. The decrease in intensity is primarily due to the excitonic interactions of the heme groups. The decreased peak value at the 1399 cm^−1^ band indicated that intermolecular distances of the heme groups dramatically increased during light therapy, which facilitated energy migration in the form of electronic transitions on the porphyrin network. The intensity of the O_2_ binding marker band at 1639 cm^−1^ had decreased in the first 10 min, which was the O_2 _binding marker. LED irradiation led to the R-to-T state transition in irradiated Hb. During this short duration, the O_2_-binding ability of Hb was not enhanced. When the irradiation time was prolonged from 5 to 30 min, the intensity of the 1639 cm^−1^ band was gradually increased, implying that the T-to-R state transition of irradiated Hb occurred. The prolongation of the irradiation time (from 5 to 30 min) led to an increase in the oxygenated Hb content during this period. Abundant LED irradiation promoted Hb to combine with O_2_ with the prolonged irradiation time. The increased peak intensity at marker bands of oxygenated Hb showed that O_2_ replaced H_2_O after a prolonged irradiation time, meaning that irradiation promoted the oxygen-transporting ability of Hb. Previous studies also found that it could lead to an increase in the velocity of blood microcirculation [41]. The decrease of the band at 1399 cm^−1^ after 30 min of irradiation is that for this prolonged irradiation period the light first unfolds the deoxy Hb and then photodamages the formed heme aggregation.

**Figure 6:**
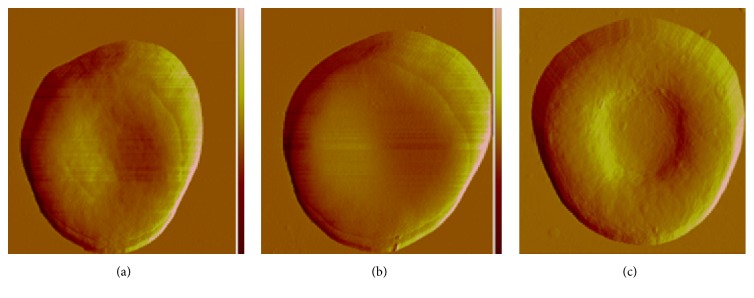
AFM data of the erythrocytes ((a) erythrocyte from hypertension person after irradiation, (b) erythrocyte from hypertension person before irradiation, and (c) erythrocyte from healthy person).

**Figure 7:**
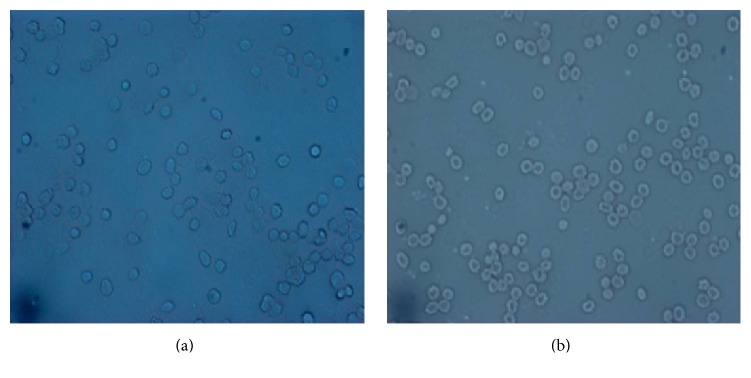
Microscopic images of red light irradiated and nonirradiated erythrocyte from hypertension person.

In Figure 6, a low magnification (10 × 10 *μ*m^2^square scanning) view of both untreated and irradiated whole erythrocytes showed an image of a disk with a central depression and the diameters of the two kinds of cell were both approximately 8 *μ*m. However, AFM image details indicated that there are some differences between the erythrocyte of hypertension after irradiation (a) and erythrocyte of hypertension (b). And the erythrocyte of hypertension after irradiation (a) looks close to normal person erythrocyte (c) in terms of the morphology.  Figure 7 showed that the LED irradiation treated hypertension erythrocytes (30 min) did not show significant cellular morphology change/damage after 48 hours of culture. Therefore, this strategy could be considered as a safe method.

## 6. Conclusions

In this study, the red light LED irradiation effect on erythrocyte and electronic-conformational interactions in Hb molecules has been studied experimentally. We studied the molecular changes of irradiated Hb isolated from patients with hypertension components. Results confirmed that (I) the 1399 cm^−1^ band, which is the marker for heme aggregation, decreased gradually with prolonged irradiation time. It offers evidence supporting heme aggregation in response to photoinduced denaturation of Hb and demonstrated that LED irradiation could inhibit heme aggregation. (II) *ν*_10_ band at 1639 cm^−1^ is known as a marker band for O_2_ concentration. During the period of LED irradiation, the extent of deoxygenation initially decreased and then rapidly increased with prolonged irradiation time. (III) The bands at 1357, 1548, and 1605 cm^−1^ are marker bands of deoxygenated Hb, while the bands at 1376, 1585, and 1639 cm^−1^ are the characteristic bands of oxygenated Hb. The variation in the intensities indicates that, after 30 min of LED irradiation, the oxygenated Hb content would exceed the controlled Hb content during the light therapy process. (IV) The variance of intensities at the 1399 cm^−1^ and 1639 cm^−1^ bands showed that, in our study, the suitable LED irradiation time was 30 min with a power setting of 15.0 mW. Experimental data showed that red LED phototherapy is feasible and effective at inhibiting heme aggregation and enhanced the HbO_2_ content* in vitro*. It provides persuasive experimental data in support of treating hypertension* in vivo*.

## Figures and Tables

**Table:** (a) Electrical-Optical Characteristics at *I*_*F*_ = 400 mA, Ta = 25°C

Parameter	Symbol	Min	Type	Max	Unit
Luminous	Φ_*v*_	30	~	40	lm
Wavelength	*λ*_*D*_	640	~	660	nm
Forward voltage	*V*_*F*_	2.0	~	2.6	V
Power dissipation	*P*_*D*_	0.80	~	1.04	W
View angle	2*θ*_1/2_	~	60	~	deg.
Thermal resistance	*Rθ*_*J*−*B*_	~	12	~	°C/W

**Table:** (b) Absolute Maximum Ratings

Parameter	Symbol	Value	Unit
Forward Current	*I*_*F*_	400	mA
Junction Temperature	*T*_*j*_	115	°C
Operating Temperature	*T*_opr_	−40~+60	°C
Storage Temperature	*T*_stg_	0~+60	°C
ESD sensitivity	~	±2,000 V HBM	~
Temperature Coefficient of voltage	~	−5	Mv/°C
DC Pulse Current (@ 1 KHz, 10% duty cycle)	*I*_FP_	1000	mA
Reverse Voltage	*V*_*R*_	Not designed for reverse operation	

*∗*Notes

1. Tolerance of Luminous Flux is ±3%.

2. Tolerance of Forward Voltage is ±0.1 V.

**Table 2:** Exquisite assignment of normal hemoglobin Raman bands from healthy person.

Frequency (cm^−1^)	Mode	Local coordinate	Symmetry
677	*ν*_7_	*δ*(pyr deform)_sym_	A_1g_
755	*ν*_15_	*ν*(pyr breathing)	B_1g_
1001	Phe	*ν*(C_*β*_C_1_)	
1120	*ν*_22_	*δ*(CH_2_) twisting, wagging	A_2g_
1172	*ν*_30_	*ν*(pyr half-ring)_asym_	B_2g_
1213	*ν*_5_ + *ν*_18_	*δ*(C_*m*_H)	B_1g_ or E_u_
1226	*ν*_13_	*δ*(C_*m*_H)	B_1g_
1303	*ν*_21_	*δ*_asym_(C_*m*_H)	A_2g_
1357	*ν*_4_	*ν*(pyr half-ring)_sym_	A_1g_
1376	*ν*_4_	*ν*(pyr half-ring)_sym_	A_1g_
1399	*ν*_20_	*ν*(pyr quater-ring)	A_2g_
1427	*ν*_28_	*ν*(C_*α*_C_*m*_)_sym_	B_2g_
1548	*ν*_11_	*ν*(C_*β*_C_*β*_)	B_1g_
1585	*ν*_37_	*ν*(C_*α*_C_*m*_)_asym_	E_u_
1605	*ν*_19_	*ν*(C_*α*_C_*m*_)_asym_	A_2g_
1639	*ν*_10_	*ν*(C_*α*_C_*m*_)_asym_	B_1g_
